# Effects of a sucralfate-containing ointment on quality of life and symptoms associated with hemorrhoidal disease: patient-reported results of a Slovakian, pharmacist-led observational survey

**DOI:** 10.3389/fgstr.2023.1213433

**Published:** 2023-09-05

**Authors:** Miroslava Snopková, Ondrej Sukel‘, Jan Micanko

**Affiliations:** ^1^ Department of Organization and Management in Pharmacy, Faculty of Pharmacy, Comenius University Bratislava, Bratislava, Slovakia; ^2^ Slovak Chamber of Pharmacists, Bratislava, Slovakia; ^3^ Allio Ltd., Šamorín, Slovakia

**Keywords:** clinical pharmacist, community pharmacy, hemorrhoidal disease, minor disease, quality of life, sucralfate ointment

## Abstract

**Purpose:**

This pharmacist-led study evaluated the effect of a rectal ointment containing sucralfate on quality of life, symptom frequency and time to relief of symptoms in Slovakian individuals with hemorrhoidal disease (HD).

**Methods:**

The multicenter prospective survey was conducted at 45 community pharmacies in Slovakia. Pharmacists invited adults (≥18 years) using sucralfate-containing ointment for their HD-related symptoms to participate.

**Results:**

241 patients completed the HEMO-FISS-QoL questionnaire and a survey of symptom frequency at the beginning and end of the 14-day survey period. The primary endpoint was the change in HEMO-FISS-QoL scores in patients with hemorrhoidal symptoms during the 7 days before the initial pharmacy visit. Of the 241 patients enrolled in the survey, 144 had experienced hemorrhoidal symptoms within the preceding 7 days (mean age 51 years; 59.0% female). For these 144 patients, the total HEMO-FISS-QoL score decreased (i.e., quality of life was improved) from baseline by a mean of –8.7 (95% confidence interval –12.6, –6.2; P<0.001) at day 14. The frequency of hemorrhoidal symptoms was significantly reduced (P<0.001 vs baseline). Symptom relief was rapid; at 1-hour post-treatment 54.6% of patients had relief from pain and 56.3% from itching, and by 24 hours post-treatment most patients had relief from these symptoms (77.2% and 73.0%, respectively). No incidents nor adverse events related to sucralfate-containing ointment were reported to pharmacists.

**Conclusion:**

The results of this pharmacist-led observational survey suggest that the sucralfate-containing ointment could improve quality of life in patients with HD, providing rapid relief with a good safety profile. To confirm these results in a larger, well-defined patient population, randomized controlled trials in patients with clinically diagnosed HD are warranted.

## 1 Introduction

Hemorrhoidal disease (HD) is a condition affecting the anal cushions, potentially causing pain, anal bleeding, discomfort, itching, swelling, rectal prolapse, soiling, and fecal incontinence (1, 2). Although it is common, most patients have low-grade disease and mild symptoms, and do not seek medical advice, so the exact prevalence is not well known (2). A recent international web-based survey conducted in a representative sample of the general adult population from eight countries in Europe and South America demonstrated that 11% of respondents had symptoms of HD (1). Only 40% of patients with HD seek treatment from their doctor as a first step; most will try to find an effective treatment through their own research, talking to friends, or seeing a pharmacist (1).

Low-grade HD can usually be managed conservatively using lifestyle changes (e.g., hydration, dietary changes, and fiber supplementation) to reduce constipation and improve bowel habits, as well as topical ointments or phlebotonic agents to relieve symptoms (3, 4). These treatments are commonly available from pharmacies, and according to an international survey of patients, approximately 70% of patients with HD used topical ointments as their first treatment step (1).

Despite the widespread use of topical treatments, there are limited data on the effectiveness of many of these agents (5–7). A rectal ointment containing 3% sucralfate (Emotralex^®^, manufactured by Egis Pharmaceuticals PLC; hereafter referred to as ‘sucralfate-containing ointment’), a class IIa medical device, became available in Slovakia in 2020 for the treatment of symptoms associated with HD and its complications (e.g., eczema and anal fissures). When the ointment is applied to inflamed, itchy skin, it covers and protects the epidermis and provides care to the affected skin promoting skin regeneration. It decreases the drying out of the skin, improves wound healing, and reduces the risk of fissure and injury caused by defecation.

Previous observational research in Italy (the EMOCARE survey) indicated that this sucralfate-containing ointment improved quality of life (QoL) in patients with HD (8). The aim of the present LEONIDAS survey was to provide additional data on the effect of the ointment on QoL, symptom frequency, and time to onset of symptom relief in Slovakian individuals seeking treatment for HD at community pharmacies.

## 2 Patients and methods

This pharmacist-led, multicenter, observational, prospective patient survey was conducted at 45 community pharmacies in Slovakia between December 3, 2020 and March 31, 2021, in association with Allio ltd., a contract research company. Pharmacists underwent training with Allio Ltd., to aid in their identification of eligible patients. These were individuals aged ≥18 years who were seeking a local treatment for hemorrhoidal symptoms and who had chosen to use the sucralfate-containing ointment for these symptoms, with or without other hemorrhoidal treatments, after a discussion of treatment options with the participating pharmacist.

The assessment of the effect of the sucralfate-containing ointment on QoL, symptoms, and ease of use was undertaken in those patients who had been experiencing symptoms within the 7 days prior to visiting the pharmacy (QoL cohort). Because the study was conducted during the severe acute respiratory syndrome coronavirus 2 (SARS-CoV-2) pandemic, this 7-day period allowed for inclusion of any patients with recent, but not necessarily current, symptoms who may have been prevented from attending the pharmacy promptly because of local pandemic control regulations.

On the day that treatment was sought at the pharmacy (which was also the screening and enrolment day, i.e., Day 0), the pharmacist explained the study to patients and, for those willing to participate, obtained their verbal and written informed consent to take part in the research. The pharmacist then collected information from each patient on their age, sex, hemorrhoidal anamnesis (i.e., anal complaints and constipation), and concomitant treatments for HD, and advised them how to use the sucralfate-containing ointment in accordance with the approved instructions for use (9). The ointment was sold or provided to patients as per usual practice for over-the-counter prescriptions by the pharmacists. Patients were advised to apply the ointment around the anus or insert small quantities into the rectum using the applicator once or twice daily (depending on the severity of symptoms) for approximately 14 days, or until symptoms resolved. Use of the sucralfate-containing ointment was discouraged if patients had bleeding hemorrhoids, although they could have spotting. Patients were advised to consult a doctor if symptoms did not improve within 1–2 weeks, as per the instructions for use (9).

To assess QoL, patients in the QoL cohort completed the Slovakian version of the validated Hemorrhoidal Disease and Anal Fissure Quality of Life (HEMO-FISS-QoL) questionnaire (10) on Day 0 and Day 14 (end of study). This paper questionnaire, which was filled out by the patients, contains 23 items in four QoL domains (i.e., physical disorders, psychology, defecation, and sexuality); for each question, patients ranked their response on a 5-point Likert scale, from 1 (never) to 5 (always), where a higher score represents worse QoL (range of scores: 0–100). For each question, there was also a sixth option: not applicable.

Pseudonymized patient information, survey responses, and HEMO-FISS-QoL questionnaire responses were recorded by the pharmacist in an electronic case report form (eCRF) and held by Allio Ltd. in a secure web-based application in accordance with local privacy regulations. Any paper-based information recorded by the pharmacist was destroyed after completion of the eCRF. Only anonymized data were available to the authors and sponsor. In Slovakia, ethics committee approval is not mandatory under national legislation for surveys, and as such, no approval was requested. This study was conducted in accordance with the Helsinki Declaration of 1964 and its later amendments, and all patients provided written informed consent for personal data processing. No patient data were kept by the pharmacists upon completion of the survey.

The pharmacist conducted a telephone follow-up on Day 2. All patients were asked to report any incidents or risk of incidents, including adverse events or special situations they may have experienced. Patients in the QoL cohort were also questioned about their experience in applying the sucralfate-containing ointment and about the time to onset of symptom relief (30 minutes or 1, 12, 24, or 48 hours after the first application of the ointment).

On Day 14, patients were followed up by telephone or during a visit to the pharmacy. The pharmacist recorded whether the patient was still using the sucralfate-containing ointment, the frequency of their hemorrhoidal symptoms, their experience with applying the sucralfate-containing ointment, any incidents or risk of incidents, including adverse events or special situations occurring in relation with the sucralfate-containing ointment during the survey, and the self-reported patient responses to the HEMO-FISS-QoL questionnaire.

The primary study endpoint was the change in overall HEMO-FISS-QoL, and secondary endpoints were symptom frequency and time to symptom relief. Both primary and efficacy endpoints, as well as ease of use of the sucralfate-containing ointment, were assessed in the QoL cohort.

### 2.1 Statistical analysis

Sample size was not determined *a priori*. The variables were analyzed using descriptive statistics, reported as frequency for categorical variables and mean or median, standard deviation (SD) or 95% confidence intervals (CIs), and range for continuous variables. When data were missing for individual or total HEMO-FISS-QoL domain scores because the ‘not applicable’ option had been chosen, the score for that item was imputed using the average value of the population participating in the survey. Given the non-normal distribution of HEMO-FISS-QoL score, the change from baseline was analyzed using Wilcoxon’s rank sign test. The distribution of patients according to their category of frequency of hemorrhoidal symptoms (i.e., never, rarely, sometimes, very often, or always) was compared at Day 0 and Day 14 using the Pearson’s chi-squared test. A *P* value of <0.05 was considered statistically significant. Statistical analysis was performed using the SPSS statistical processing software version 20.1 (IBM Corp.; Armonk, NY).

## 3 Results

### 3.1 Patient demographics and baseline characteristics

Overall, 241 patients were enrolled at 45 pharmacies, of whom 237 (98.3%) participated in the Day 14 follow-up visit. Patients were aged between 19 and 92 (mean [SD] 51.3 [15.4]) years, and 55.6% (*n* = 134) were female (**Table 1**). Concurrent constipation was reported by 61/241 patients (25.3%). Just over one-third (*n* = 86, 35.7%) of the patients had consulted a doctor for HD and 7.5% (*n* = 18) were using laxatives. Of the 86/241 patients (35.7%) who were using systemic treatment for HD, 63 (73.3%) were taking micronized purified flavonoid fraction.

**Table 1:** Baseline demographic and clinical characteristics of all patients.

Characteristic	All patients (*N* = 241)
Female, *n* (%)	134 (55.6)
Age, years	
Mean ± SD	51.3 ± 15.4
Median (range)	50.0 (19–92)
Concurrent constipation, *n* (%)	
Never	157 (65.1)
<18 months	34 (14.1)
19 months to 5 years	11 (4.6)
>5 years	16 (6.6)
Don’t know	23 (9.5)
Consulted a doctor for HD, n (%)	86 (35.7)
Laxative use, *n* (%)	18 (7.5)
Systemic hemorrhoid treatment, *n* (%)	86 (35.7)

HD, hemorrhoidal disease; SD, standard deviation.

The QoL cohort comprised 144 patients (i.e., patients who at the time of enrolment had hemorrhoidal symptoms or who had experienced these in the 7 days prior to the study). Compared with the overall cohort, the QoL cohort included higher proportions of women (*n* = 85; 59.0%), patients who had consulted a doctor about HD (*n* = 70; 48.6%), and patients using laxatives (*n* = 14; 9.7%; **Table 2**). Within this cohort, 52 patients (36.1%) were using systemic treatment for HD, including micronized purified flavonoid fraction (*n* = 35; 67.3%). Almost the entire QoL cohort (*n* = 142; 98.4%) participated in the Day 14 visit (i.e., the last follow-up), which was by telephone in most patients (*n* = 121; 85.2%); only 21 patients (14.8%) completed the Day 14 follow-up by an in-person pharmacy visit.

**Table 2:** Baseline demographic and clinical characteristics of patients in the quality of life (QoL) cohort.

Characteristic	Patients in QoL cohort (*n* = 144)
Female, *n* (%)	85 (59.0)
Age, years	
Mean ± SD	50.9 ± 15.8
Median (range)	50 (19–92)
Concurrent constipation, *n* (%)	
Never	98 (68.0)
<18 months	16 (11.1)
19 months to 5 years	6 (4.2)
>5 years	9 (6.3)
Don’t know	15 (10.4)
Consulted a doctor for HD, *n* (%)	70 (48.6)
Laxative use, *n* (%)	14 (9.7)
Systemic hemorrhoid treatment, *n* (%)	52 (36.1)

HD, hemorrhoidal disease; SD, standard deviation.

### 3.2 Quality of life

**Figure 1:**
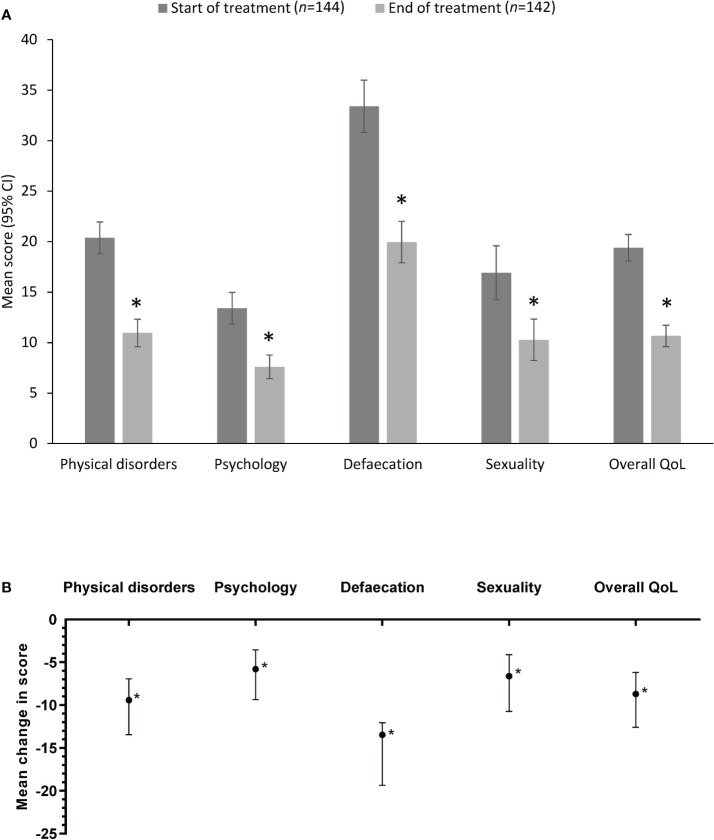
HEMO-FISS-QoL scores in the quality of life cohort **(A)** at Day 0 and Day 14 (mean values) and **(B)** as the mean difference between Day 0 and Day 14. Error bars represent 95% CI. A reduction in score indicates an improvement. *P<0.001 vs start of treatment. CI, confidence interval; HEMO-FISS-QoL, Hemorrhoidal Disease and Anal Fissure Quality of Life questionnaire; QoL, quality of life.

QoL data were available from all 144 patients in the QoL cohort at baseline (Day 0) and from 142 of these patients at the Day 14 follow-up. The overall mean score and individual domain scores at Day 0 and Day 14 are shown in **Figure 1A**. The overall mean HEMO-FISS-QoL score and the mean score for each individual domain of the scale improved significantly from baseline to the end of treatment (**Table 3**, **Figure 1B**). The overall mean score decreased from 19.4 at Day 0 to 10.7 at Day 14, corresponding to an improvement of 45% (change from baseline in overall score of −8.7; 95% CI −12.6, −6.2). Defecation was the domain with the highest QoL score at Day 0, as well as the domain with the largest improvement at Day 14, with a mean change from Day 0 in score of −13.5 (95% CI −19.4, −12.1; **Table 3**).

**Table 3:** Change from baseline in the HEMO-FISS-QoL scores in the quality of life (QoL) cohort.

Domain	HEMO-FISS-QoL scores				*P*-value^1^
	Day 0 (*n* = 144)	Day 14 (*n* = 142)	Change from Day 0 at Day 14		
	Mean (95% CI)	Mean (95% CI)	Mean (95% CI)	Percentage	
Individual QoL domains					
Physical disorders	20.4 (18.8, 22.0)	11.0 (9.6, 12.3)	–9.4 (–13.4, –7.0)	−46%	<0.001
Psychology	13.4 (11.8, 15.0)	7.6 (6.4, 8.8)	–5.8 (–9.4, –3.6)	−43%	<0.001
Defecation	33.4 (30.8, 36.0)	20.0 (17.9, 22.0)	–13.5 (–19.4, –12.1)	−40%	<0.001
Sexuality	16.9 (14.3, 19.6)	10.3 (8.2, 12.3)	–6.6 (–10.8, –4.1)	−39%	<0.001
**Overall QoL**	19.4 (18.1, 20.7)	10.7 (9.6, 11.7)	–8.7 (–12.6, –6.2)	−45%	<0.001

CI, confidence interval; HEMO-FISS-QoL, Hemorrhoidal Disease and Anal Fissure Quality of Life questionnaire.

^1^Wilcoxon’s rank sign test of the mean change from baseline in QoL score.

### 3.3 Impact on symptoms

**Figure 2:**
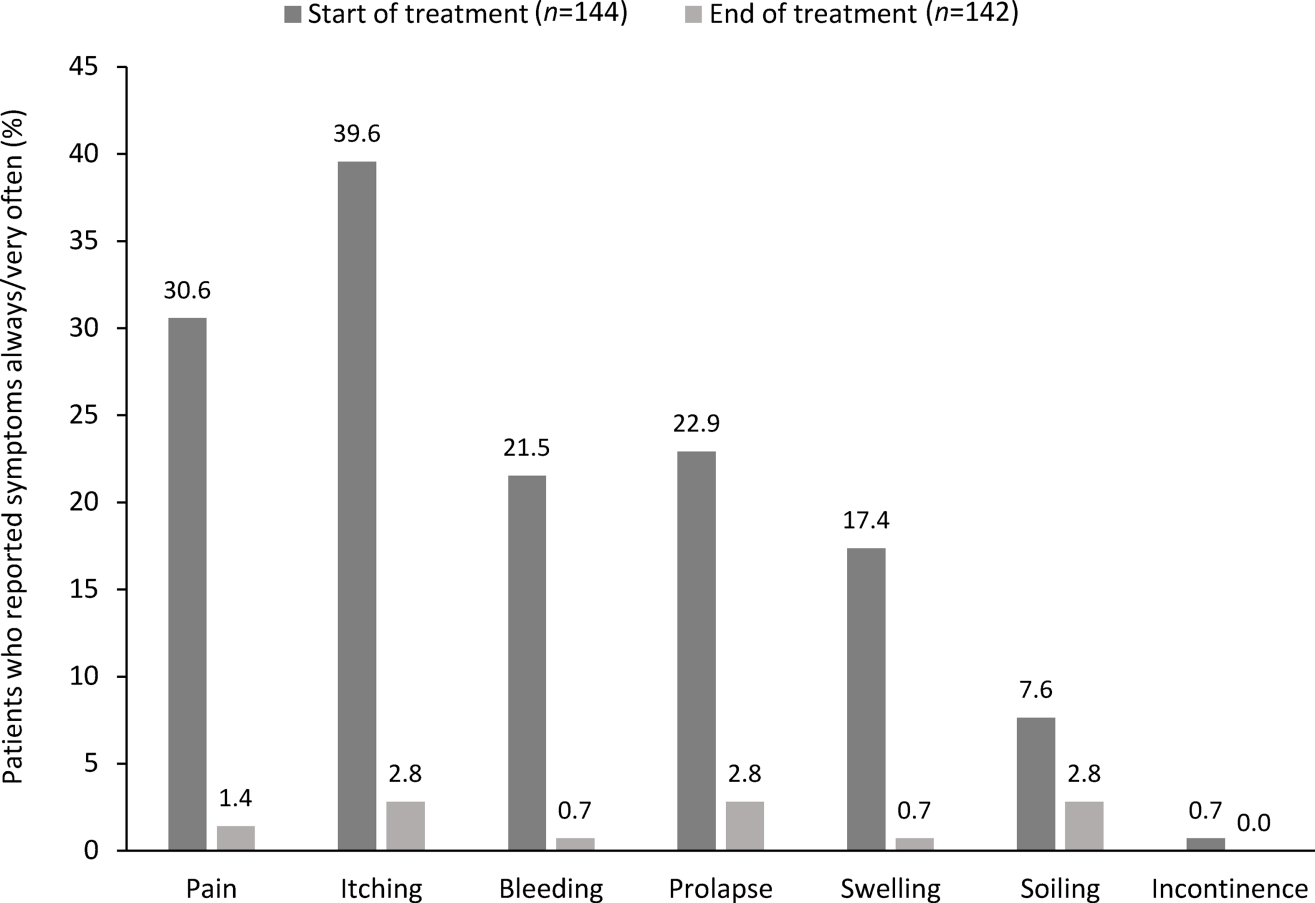
Change in the percentage of patients reporting symptom frequency as ‘very often’ or ‘always’ at Day 0 and Day 14 in the quality of life cohort.

In the QoL cohort (*n* = 144), hemorrhoidal symptoms reported at Day 0 were pain (*n* = 127; 88.2%), itching (*n* = 126; 87.5%), bleeding (*n* = 112; 77.8%), swelling (*n* = 104; 72.2%) and prolapse (*n* = 104; 72.2%). In addition, 50.0% of patients (*n* = 72) experienced soiling and 18.1% (*n* = 26) experienced fecal incontinence. The proportions of patients who experienced these symptoms ‘always’ or ‘very often’ at Day 0 and at Day 14 are shown in **Figure 2**. At Day 14, the proportion of patients experiencing each of these symptoms had decreased significantly (**Table 4**).

**Table 4:** Hemorrhoidal symptoms at Day 0 and Day 14 in the quality of life cohort.

Symptom^1^	Never, *n* (%)		Rarely, *n* (%)		Sometimes, *n* (%)		Very often, *n* (%)		Always, *n* (%)		*P*-value^2^
	Day 0 (*n* = 144)	Day 14 (*n* = 142)	Day 0 (*n* = 144)	Day 14 (*n* = 142)	Day 0 (*n* = 144)	Day 14 (*n* = 142)	Day 0 (*n* = 144)	Day 14 (*n* = 142)	Day 0 (*n* = 144)	Day 14 (*n* = 142)	
Pain	17 (11.8)	99 (69.7)	33 (22.9)	33 (23.2)	50 (34.7)	8 (5.6)	34 (23.6)	2 (1.4)	10 (6.9)	0	<0.001
Itching	18 (12.5)	101 (71.1)	33 (22.9)	24 (16.9)	36 (25.0)	13 (9.2)	45 (31.3)	4 (2.8)	12 (8.3)	0	<0.001
Bleeding	32 (22.2)	113 (79.6)	40 (27.8)	22 (15.5)	41 (28.5)	6 (4.2)	21 (14.6)	1 (0.7)	10 (6.9)	0	<0.001
Prolapse	40 (27.8)	105 (73.9)	31 (21.5)	16 (11.3)	40 (27.8)	17 (12.0)	21 (14.6)	3 (2.1)	12 (8.3)	1 (0.7)	<0.001
Swelling	40 (27.8)	109 (76.8)	44 (30.6)	28 (19.7)	35 (24.3)	4 (2.8)	16 (11.1)	1 (0.7)	9 (6.3)	0	<0.001
Soiling	72 (50.0)	127 (89.4)	44 (30.6)	10 (7.0)	17 (11.8)	4 (2.8)	10 (6.9)	1 (0.7)	1 (0.7)	0	<0.0001
Incontinence	118 (81.9)	139 (97.9)	21 (14.6)	3 (2.1)	4 (2.8)	0	0	0	1 (0.7)	0	<0.0001

^1^At the Day 0 visit, patients were asked to indicate which of these hemorrhoidal symptom(s) they had experienced over the prior 7 days, and to indicate the frequency of each over that time period; ‘never’ meant ‘not at all the prior 7 days’, although this was not explicitly explained to the patients. At the Day 14 follow-up visit, it was first established whether patients were still experiencing hemorrhoidal symptoms (yes/no question), and if so, to indicate which symptom(s) and at what frequency. While not actually specified, ‘never’ at this follow up meant the symptom was absent on Day 14.

^2^P-values compared Day 14 versus Day 0 for all symptoms and frequency.

**Figure 3:**
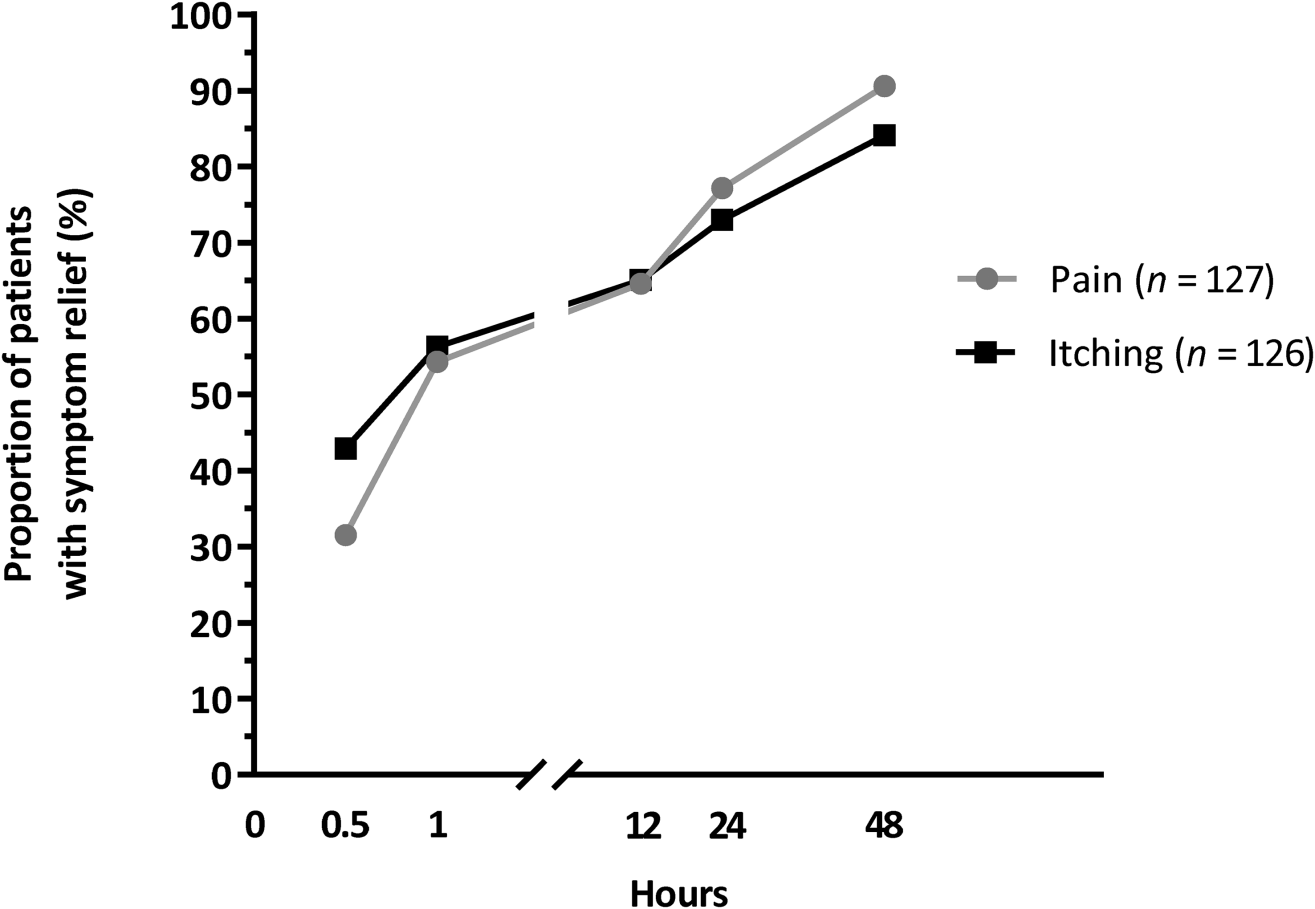
The percentage of patients reporting relief from pain and itching at each time point after starting treatment in the quality of life cohort.

The symptoms that were relieved most quickly after application of the sucralfate-containing ointment included the two most common symptoms, pain and itching, as well as bleeding and swelling (**Table 5**). Approximately one in two patients gained relief from pain and itching within 1 hour of ointment application (54.3% [*n* = 69/127] for pain and 56.3% [*n* = 71/126] for itching; **Figure 3**). At 24 hours after starting the sucralfate-containing ointment, 77.2% (*n* = 98/127) and 73% (*n* = 92/126) of patients reported relief from pain and itching, respectively. The corresponding proportions reporting relief of these symptoms were 90.6% (*n* = 115/127) and 84.1% (*n* = 106/126), respectively, at 48 hours after starting sucralfate-containing ointment (**Figure 3**).

**Table 5:** Time to symptom relief in the quality of life cohort.

Timing of relief, *n* (%)	Pain (*n* = 127)	Itching (*n* = 126)	Bleeding (*n* = 112)	Prolapse (*n* = 104)	Swelling (*n* = 104)	Soiling (*n* = 72)	Incontinence (*n* = 26)
30 minutes	40 (31.5)	54 (42.9)	23 (20.5)	2 (1.9)	15 (14.4)	9 (12.5)	5 (19.2)
1 hour	29 (22.8)	17 (13.5)	13 (11.6)	9 (8.7)	14 (13.5)	1 (1.4)	0
12 hours	13 (10.2)	11 (8.7)	12 (10.7)	17 (16.3)	21 (20.2)	5 (6.9)	0
24 hours	16 (12.6)	10 (7.9)	12 (10.7)	18 (17.3)	21 (20.2)	13 (18.1)	1 (3.8)
2 days	17 (13.4)	14 (11.1)	16 (14.3)	19 (18.3)	18 (17.3)	7 (9.7)	5 (19.2)
No relief in 2 days	12 (9.4)	20 (15.9)	36 (32.1)	39 (37.5)	15 (14.4)	37 (51.4)	15 (57.7)

### 3.4 Ease of use

More than 95% (*n* = 136/142) of patients found the application of the sucralfate-containing ointment to be ‘very easy’, ‘easy’ or ‘neither easy nor hard’ to use at Day 2 and Day 14 (**Table 6**).

**Table 6:** Ease of use of the sucralfate-containing ointment in the quality of life cohort.

Category	Ease of use, *n* (%)	
	Day 2 (*n* = 144)	Day 14 (*n* = 142)
Very easy	50 (34.7)	47 (33.1)
Easy	37 (25.7)	39 (27.5)
Neither easy nor difficult	52 (36.1)	50 (35.2)
Difficult	1 (0.7)	2 (1.4)
Very difficult	4 (2.8)	4 (2.8)

### 3.5 Treatment exposure

The mean ± SD treatment duration was 11.9 ± 0.6 (range 1–14) days, and the mean ± SD number of applications was 1.66 ± 0.5 times a day in the QoL cohort.

### 3.6 Safety

None of the patients in the overall cohort (*n* = 144) reported experiencing any incidents or risk of incidents, including adverse events or special situations in relation with the sucralfate-containing ointment or other products during the survey.

## 4 Discussion

To our knowledge, this is the first pharmacist-led study investigating the effects of a sucralfate-containing ointment in a routine clinical practice setting in Slovakia. This prospective study conducted across 45 Slovakian clinical pharmacies confirmed that the sucralfate-containing ointment significantly improved QoL and reduced both overall symptoms, and symptoms experienced within the last 7 days, in patients with HD.

In this survey, ‘defecation’ was the most severely affected QoL domain among patients with HD, consistent with data from the Italian EMOCARE survey (8). That study also used the HEMO-FISS-QoL questionnaire to assess QoL, and found a significant reduction in total scores (i.e., an improvement), as well as in each individual QoL domain, after using sucralfate-containing ointment for 14 days (8), which is consistent with our finding for use of the treatment over approximately 12 days. In both the Italian EMOCARE survey and our study, the greatest improvement (reduction in score from baseline) was seen in the most affected domain (i.e., ‘defecation’).

The characteristics of the Slovakian patients with HD in our study were consistent with the known epidemiology of the disease. The peak age for HD occurrence is between 45 and 65 years (1, 11–13), and the median age of patients in our study was 50 years. The number of women with HD slightly exceeded that of men in our study, which has also been reported by some researchers (1, 13), but not others (11, 12). However, this may simply reflect a greater willingness by women to seek healthcare compared with men (14). Additionally, it could be explained by the history of pregnancies among women, pregnancies being a risk factor for HD.

The Italian EMOCARE survey reported that almost half of its patients with HD had constipation (1) and 21.4% used laxatives, whereas only about 25% of patients in the current study reported constipation and 7.5% used laxatives. Similarly, a higher proportion of EMOCARE patients (52.4%) had consulted a physician for HD compared with Slovakian patients in the current study (35.7%). These discrepancies suggest differences between the two countries in healthcare-seeking behavior by patients with HD, potentially due to different healthcare systems or patient attitudes. They may also be explained by the SARS-CoV-2 pandemic that limited patient access to physicians, or by the fact that patient responses were recorded by a third-party (pharmacist). Pharmacists recording patient responses and knowing intimate details of the disease may have biased how the patients responded to the questionnaire, in turn diminishing the reported severity of the symptoms. Irrespective of the differences, our data and the EMOCARE results are both consistent with previous research, showing that patients with HD often do not seek, or delay seeking, treatment from doctors (1, 15, 16). Individuals with HD commonly cite embarrassment or shame as key reasons for not seeking medical care (16).

Despite some differences in the incidences of symptoms, our data are generally consistent with previous research, in that pain and itching are among the most common self-reported symptoms of HD (1, 2). Pain was the most common symptom reported by patients in the current study (affecting 88.2% of patients) and in the Italian EMOCARE survey (82.8% of patients) (8). The second most common symptom in the current study was itching (affecting 87.5%), whereas swelling was the second most common symptom in the EMOCARE survey (affecting 82.4%); itching was reported by 68.6% of Italian patients (8). In contrast, itching was reported by only 35% of the 1725 patients in an international web-based survey (1). The difference in the prevalence of itching between the web-based survey (35.1%) (1) and the two pharmacy-based surveys (68.6% (8) and 87.5%) suggests that itching may be a symptom that prompts patients to seek treatment, a conclusion that is supported by qualitative research on patients’ experience of HD (16).

Importantly, our data show rapid relief of both pain and itching during treatment with the sucralfate-containing ointment, with more than 50% of patients reporting relief from these symptoms within 1 hour of applying the ointment. Moreover, 77% of patients reported relief of pain at 24 hours post-treatment initiation. The pain-relieving properties of a sucralfate-containing ointment (10% sucralfate in a petrolatum base) have been previously demonstrated in a randomized comparison with lidocaine ointment used postoperatively after hemorrhoidectomy (17). In that study, pain relief was significantly better with the sucralfate-containing ointment than lidocaine ointment on postoperative Days 1, 3, and 7 (17).

Prolapse was reported more frequently in the current study (72.2%) than in the Italian EMOCARE survey (43.8%) (8), the international web-based survey (15%) (1), and the international Chronic venous and HemORrhoidal diseases evaluation and Scientific research (CHORUS) study (36.2%) (2). The prevalence of prolapse in our study was unexpectedly high, but could not be confirmed because symptoms were self-reported, and patients did not undergo physical examination. It is possible that patients did not fully understand what the term meant or were mistaking swelling for prolapse. The true prevalence of prolapse among Slovakian patients with HD warrants further investigation.

The data from this study and previous research provide reassurance that sucralfate-containing ointments can improve QoL and rapidly relieve symptoms in patients with HD. In addition, patients found the treatment easy to use. While prolonged use of topical treatments can cause local reactions or skin irritation (7), none of the patients in the current study reported adverse effects to the pharmacists, indicating that 12 days of therapy with the sucralfate-containing ointment was well tolerated. Confirmation of the tolerability and safety of sucralfate-containing ointments over prolonged periods requires further investigation.

The current study was conducted during the SARS-CoV-2 pandemic, highlighting the vital role pharmacists and telepharmacy play in providing rapid and sufficient healthcare, particularly when patient access to general practitioners is limited. The early and effective management of HD relies heavily on community pharmacists assessing symptoms promptly, providing the patient with sufficient information on all treatment options, offering pharmacological advice and early intervention for rapid symptom alleviation, and providing lifestyle advice and follow-up counselling to prevent disease recurrence.

The current study has some limitations. As the survey was conducted among patients who had chosen to use this particular sucralfate-containing ointment, the study population was subject to selection bias, and patient self-reported responses were subject to response bias. No control group was included, making it difficult to draw firm conclusions about the effectiveness of the sucralfate-containing ointment. Data imputation was conducted for missing values. Additionally, as patients could take concomitant treatments, a synergistic effect could not be excluded. Furthermore, the proportion of patients using concomitant therapies with the sucralfate-containing ointment was only recorded at the start of treatment, so the impact of the concomitant medications throughout the study cannot be determined. Another limitation is that the diagnosis of HD was based on self-reported anal symptoms and could not be confirmed by physical medical examination; some patients may have misdiagnosed themselves. Of note, this study was conducted during the SARS-CoV-2 pandemic when access to doctors was limited. Since patients sought advice from community pharmacists who do not utilize clinical assessment tools such as the Goligher classification as a means to assess HD severity (given their unfamiliarity with the scale and the impossibility of performing anal examinations at community pharmacies), prolapse could not be verified. This may have constituted a significant bias in the target population to be treated with this product, and consequently, interpretation of the study results. A clearly defined population assessed and followed by physicians will be needed to confirm the product’s potential benefits.

## 5 Conclusion

The results of this pharmacist-led, Slovakian, multicenter, observational, prospective study suggest that treatment with a sucralfate-containing ointment could improve QoL and provide rapid symptom relief, is easy to use, and is safe and well tolerated in patients with symptoms of HD. Randomized controlled trials in patients with clinically-diagnosed HD would be useful to confirm these results in a larger, well-defined patient population. The role pharmacists play in the rapid and effective resolution of HD is also highlighted, particularly when patient access to general practitioners is limited.

## Data availability statement

The raw data supporting the conclusions of this article will be made available by the authors, without undue reservation.

## Ethics statement

Ethical review and approval was not required for the study on human participants in accordance with the local legislation and institutional requirements. The patients/participants provided their written informed consent to participate in this study.

## Author contributions

Conceptualization: MS and OS. Formal analysis: JM. Writing- review and editing: all authors. All authors approved the final version for submission, understand and adhere to the ICMJE criteria for authorship and had complete access to the study data. All listed collaborators are members of SLeK (Slovak Chamber of Pharmacists). All authors contributed to the article and approved the submitted version.
